# Aging Characteristics of Transformer Oil-Impregnated Insulation Paper Based on Trap Parameters

**DOI:** 10.3390/polym13091364

**Published:** 2021-04-22

**Authors:** Yanhui Wei, Wang Han, Guochang Li, Xiaojian Liang, Zhenlu Gu, Kai Hu

**Affiliations:** Institute of Advanced Electrical Materials, Qingdao University of Science and Technology, Qingdao 266042, China; wyh@qust.edu.cn (Y.W.); hanwang5438@sina.com (W.H.); 1007480177lxj@sina.cn (X.L.); guzl1997@sina.com (Z.G.); hukai17854298263@sina.com (K.H.)

**Keywords:** oil-paper insulation, pulsed electro-acoustic (PEA) method, trap parameters, molecular simulation

## Abstract

Oil-impregnated insulation paper is an important part of transformers; its performance seriously affects the life of power equipment. It is of significance to study the aging characteristics and mechanism of oil-impregnated insulation paper under thermal stress for transformer status detection and evaluation. In the work, the accelerated thermal aging was carried out at 120 °C, and DP1490, DP787, and DP311 samples were selected to represent the new, mid-aging, and late-aging status of the transformer, respectively. The space charge distribution within the specimens was measured by the pulsed electro-acoustic (PEA) method and the trap parameters were extracted based on the measurement curves. Further, the aging mechanism was studied by molecular simulation technology. A typical molecular chain defect model was constructed to study the motion of cellulose molecules under thermal stress. The experimental results show that the corresponding trap energy levels are 0.54 eV, 0.73 eV, and 0.92 eV for the new specimen, the mid-aging specimen, and the late aging specimen, respectively. The simulation results show that the trapped energy at the beginning of aging is mainly determined by the loss of H atoms. The changes in trap energy in the middle stage of aging are mainly caused by the absence of some C atoms, and the trap energy level at the end of aging is mainly caused by the breakage of chemical bonds. This study is of great significance to reveal the aging mechanism of oil-impregnated insulation paper and the modification of insulation paper.

## 1. Introduction

The power transformer is the key component of the high-voltage DC (HVDC) transmission project. The oil-impregnated paper, as the main insulation of the power transformer, plays an important role in the safe operation of electrical equipment. During the operation of the transformer, the insulation paper is prone to generating space charge due to the influence of thermal stress and other factors, which will cause local electric field distortion, aging, and breakdown of the insulation system, and poses a great threat to the safe and reliable operation of equipment [1,2,3,4,5].

In the past few decades, many scholars have made great progress in the aging problem of oil-paper insulation. The main work focuses on the aging mechanism and the characterization of aging parameters. For the former, molecular simulation technology has been used to study the aging phenomenon of oil-paper insulation. Liao et al. simulated the crystalline and amorphous regions of cellulose and further studied the interaction between cellulose and water and organic acids generated during aging [6,7]. Fujiwara et al. used the molecular dynamics model to study the conformation of a single molecular chain in vacuum and solution and analyzed the degradation process of cellulose molecules [8]. Malin et al. studied the changes of cellulose crystal density, lattice parameters, and thermal expansion coefficient at room temperature [9]. Matthews et al. used molecular simulation to study the interaction between different crystal planes of cellulose and water [10].

For the characterization of aging parameters, the mature methods for aging characterization of oil-impregnated insulation paper mainly include the DP (Degree of Polymerization) value of insulation paper, dissolved gas analysis in oil, furfural analysis, and so on. In the past several years, studies have showed that space charge and trap parameters are expected to become new methods to characterize the aging of materials, since they can reflect the micro properties of materials. Zhang et al. studied the trap energy distribution of trap on polymer insulation surface using the pulse electroacoustic (PEA) method [11]. L. Dissado et al. researched the effects of electrical aging and thermal aging on trap characteristics in XLPE (Cross Linked Polyethylene) cable insulation [12]. The author previously studied the aging model of oil-impregnated paper insulation based on the double trap level [13].

At present, the research on trap parameters of oil-paper insulation mainly focuses on the analysis of trap parameters and their effect on space charge under different aging modes. Few studies reveal the trap change mechanism from the microscopic chain movements and defects. The molecular chain of oil-paper insulation material will break apart under the action of thermal aging.

The movement and defects of the micro molecular chain are the main reasons for the change in trap parameters.

In this work, the trap parameters of oil-paper insulation under thermal aging were studied based on the experiment and molecular simulation. In the aspect of the experiment, firstly, samples with different aging degrees were prepared by accelerated aging treatment. Secondly, the micro-morphology of insulation paper with different aging degrees was observed by SEM, and the changes of acid value and micro water content of oil with different aging degrees were tested and analyzed. Further, the space charge distribution of insulation paper with different aging degrees during voltage was tested and analyzed using the PEA test system, and the trap parameters were extracted. In the aspect of molecular simulation, the molecular model of cellulose in insulation paper was constructed to study the change of orbital occupied energy of cellulose molecules with temperature. The model of aging temperature of 400 K was selected to analyze the force of the molecule and the motion state of the molecule in the simulation process. Based on this, the molecular chain defect model was constructed to study the change of affinity. Comparing the experimental results, the mechanism of the change of trap energy level of insulation paper with different aging degrees was revealed.

## 2. Materials and Methods

### 2.1. Sample Preparation

**Figure 1:**
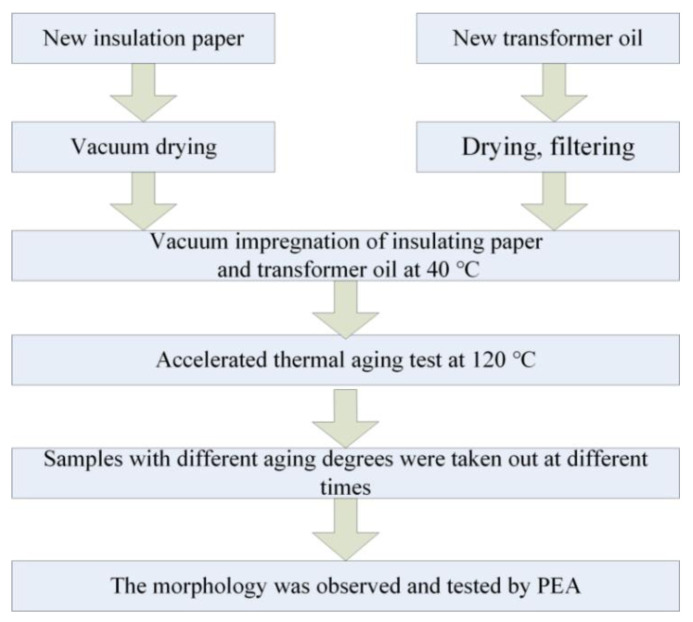
Sample preparation flow chart.

In the experiments, the insulation paper with a thickness of 0.27 mm, manufactured by Weidman company (Shanghai, China), and Karamay 25 (Xinjiang, China) mineral transformer oil, were selected. The sample preparation processes are shown in Figure 1.

Firstly, the insulation papers are pretreated by cutting and drying. The paper was cut into a shape of 8 cm × 8 cm and dried under vacuum (2XZ-1, Shanghai, China). At the same time, the oil was filtered, dried, and degassed, and then vacuum impregnated with insulation paper at 40 °C for 48 h. Secondly, the accelerated thermal aging experiment was carried out in a vacuum drying oven at 120 °C (2XZ-1, Shanghai, China). Finally, samples were taken out at 0 h, 48 h, 360 h, 720 h, and 1200 h, respectively, for subsequent testing.

The degree of polymerization (DP) was measured by the viscosity method based on the requirements in ISO 5351-2004 and GB/T1548-2004, as shown in Table 1. It is known that the degree of polymerization of new insulation paper is above 1000. With the increase in operation time, the DP of transformer insulation paper decreases slowly from 1000 to 200. When the value of DP is lower than 200, the electrical insulation and mechanical properties of insulation paper fail and cannot meet the use requirements, so it should be replaced in time [14,15].

According to the previous analysis, samples aged for 0 h (DP1490), 96 h (DP787), and 1200 h (DP311) were selected to represent the operation status of new, middle-aged, and end aged, respectively.

### 2.2. Morphological Observation

The samples were observed by scanning electron microscope (SEM, PhenomProx, Fiona company, Schinveld, The Netherlands), as shown in Table 2. It can be seen that the color of oil changes from light to dark brown over the aging time.

The cellulose of the new sample (DP1490) is thick, strong, and stretched, and the surface is smooth without wrinkles. The samples of the middle stage of aging (DP787) began to show the uneven thickness and disorderly characteristics and the surface morphology also began to appear rough. At the end of aging (DP311), the cellulose of the samples began to show the state of fracture and damage, with more wrinkles and burrs, and the fiber became more fragile and slenderer. With the decrease in DP value and the deepening of aging degree, the cellulose composition of the insulation paper gradually became fragile and slender from the initial complete strength, the surface was destroyed, and the performance stability of cellulose was also destroyed.

### 2.3. Acid Value and Micro-Water Content Analysis

**Figure 2:**
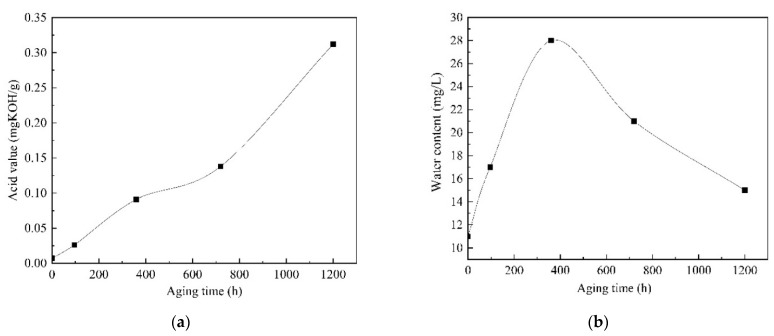
Acid value and micro-water content with aging time in the oil. (**a**) Acid value; (**b**) Water content.

The acid value and the moisture content in the oil, in relation to aging time, are shown in Figure 2. The acid value is measured by alkali burette and moisture is measured by trace moisture meter respectively. It can be seen that the acid content clearly increases with the increase in aging time due to the deterioration of the oil. From Figure 2b, it can be seen that the water content in the oil shows a trend of increasing and then decreasing with the aging time, reaching the maximum value of 28 mg/L at about 300 h of aging. During the process of aging, the acid and water in the oil promote the thermal aging rate of insulation paper and seriously affect the decrease in DP of the insulation paper.

## 3. Results and Discussion

### 3.1. Space Charge Distribution

In the experiment, the pulsed electro-acoustic method (PEA, Shanghai Heyi Electric Appliance Co., Ltd, Shanghai, China) method was adopted to measure space charge distribution in oil-impregnated paper samples with a pulse width of 5–10 ns. To better measure space charge distribution in oil-impregnated paper insulation with different aging states and avoid a breakdown during the measurement, the final test field was set at 20 kV/mm, and then the change of space charge in the sample during voltage-off was measured.

**Figure 3:**
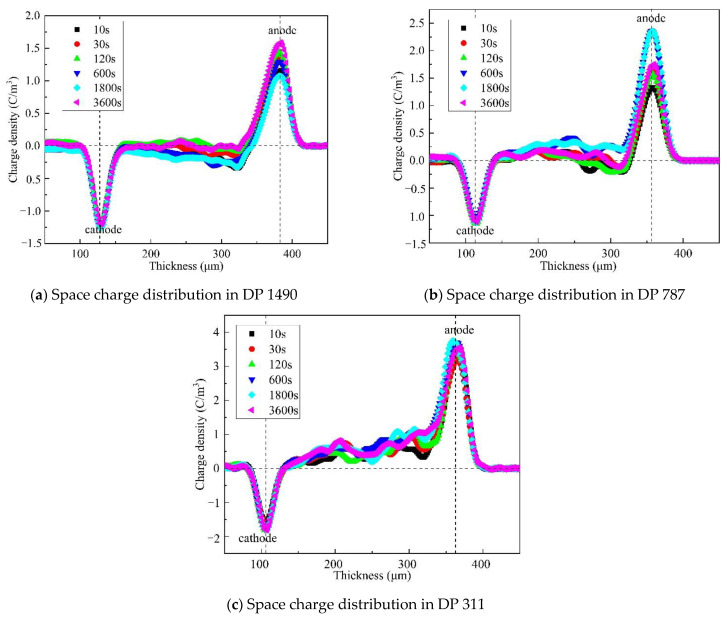
Space charge distribution of samples with different aging degrees. (**a**) DP 1490; (**b**) DP 787; (**c**) DP 311.

For each sample, space charge distribution was measured within 60 min of applying voltage and 60 min of removing voltage in a short circuit. Figure 3a shows the space charge distribution of the oil-impregnated insulation paper when it is not aged. In the early stage of voltage on (<600 s), a large amount of homopolar space charge was injected rapidly near the electrode, and then the peak charge density decreased. With the increase in time, the charge density near the electrode gradually increased and reached the peak in 600 s. With the extension of voltage-time, the peak value of charge decreased and gradually reached the steady-state. As can be seen in Figure 3a, there is a certain accumulation of negative charge near the anode at a depth of 300 μm of the sample, and this part of the charge gradually decreases to the point of disappearance under the continuous action of the applied electric field, because the sample is a two-phase composite structure material containing numerous ionizable substances [16].

It can be seen from Figure 3 that the internal charge density of the sample increases from 1.58 C/m^3^ at the initial stage of aging (DP1490) to 3.62 C/m^3^ at the end of aging (DP311), which amounts to an increase of 1.19 times. The negative charge near the anode becomes larger with the aging degree, owing to more serious aging degrees increasing the proneness to ionization.

### 3.2. Trap Parameters Analysis

The trap parameters of the oil-impregnated insulation paper with different aging stages were extracted based on the space charge distribution curves (Materials Studio, Accelrys, San Diego, CA, USA).

The amount of space charge accumulated in the sample can be expressed as follows [17,18,19,20]:

(1) $$Q(t)=\int_{0}^{d}|\rho (x,t)|sdx$$

where *Q*(*t*) is the total charge inside the medium at the moment of *t*, *ρ*(*x*,*t*) is the charge density at the moment of measurement *t* at the *x* position within the sample, *S* is the surface area of the electrode and *d* is the thickness of the sample.

After the voltage is removed, the charge density changes approximately in line with the exponential decay law.

(2) $$\sigma =Ae^{-\frac{t}{\tau }}$$

where *A* is a constant, *τ* is the decay time constant.

The relationship between trap energy level *E_t_* and current density j and trap density *N*_t_ is as follows:

(3) $$E_{t}=KT\ln(vt)$$

(4) $$j=\frac{qLKT}{2t}f_{0}(E_{t})N(E_{t})$$

where *f*_0_(*E_t_*) is the initial occupancy of the trap in the medium, and the value is 1/2, *q* is the electron quantity, 1.6 × 10^−19^ C, *k* is the Boltzmann constant, 8.568 × 10^−5^ eV/K; *T* is the absolute temperature, *K*; *v* is the electron vibration frequency, *v* = 3 × 10^12^ s^−1^; regarding the literature, the energy of the electron trap is calculated with the conduction band bottom as the zero points, and the energy of hole trap is calculated with the top of valence band as the zero points, *η*_1_ = *f*_0_(*E_t_*)*LKT*/(2*t*), *η*_2_ = *r*′*A*/*L*, *r*′ = 120 pm, *η*_1_, *η*_2_ are constants, then there are:

(5) $$N(E_{t})=\frac{\eta_{2}}{\eta_{1}}e^{-\frac{t}{\tau }}$$

From Equation (5), it can be seen that the trap parameters can be calculated by the charge decay curve of the PEA method.

**Figure 4:**
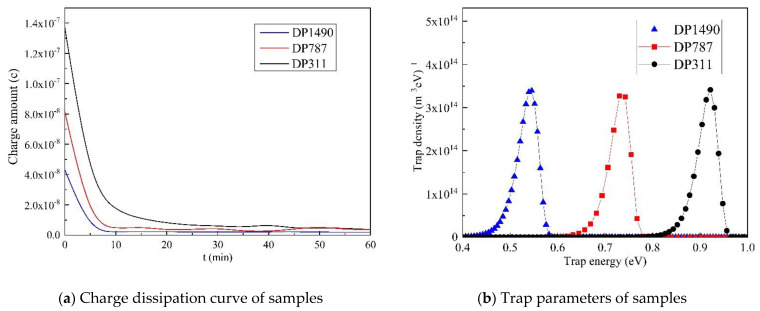
Charge dissipation and trap parameter distribution of the oil-impregnated insulation paper. (**a**) Charge dissipation curve; (**b**) Trap parameter distribution curve.

As can be seen from Figure 4a, the more serious the aging degree, the faster the charge decay rate, the greater the decay time constant, and the larger the final amount of residual charge. The trap energy level can be obtained by calculation.

It can be seen from Figure 4b that with the increase in aging time, the change of the trap density is not obvious. The trap energy levels range from 0.4 eV to 0.92 eV. It is 0.54 eV at the beginning of aging (DP1490), 0.73 eV at the middle of aging (DP787), and 0.92 eV at the end of aging (DP311). With the increase in the aging degree, the peak value gradually shifts to the right and increases from 0.54 eV to 0.92 eV, increasing by 1.7 times.

## 4. Aging Mechanism of Cellulose in Insulation Paper

Insulation paper is mainly composed of cellulose, which is a kind of chain polymer composed of glucose monomer (C_6_H_10_O_5_) with repeating units of cellobiose. Cellulose is subjected to thermal stress for a long time in transformers and is easy to change with time. This section intends to reveal the aging mechanism of cellulose by simulating the degradation process of cellulose under thermal stress.

**Figure 5:**
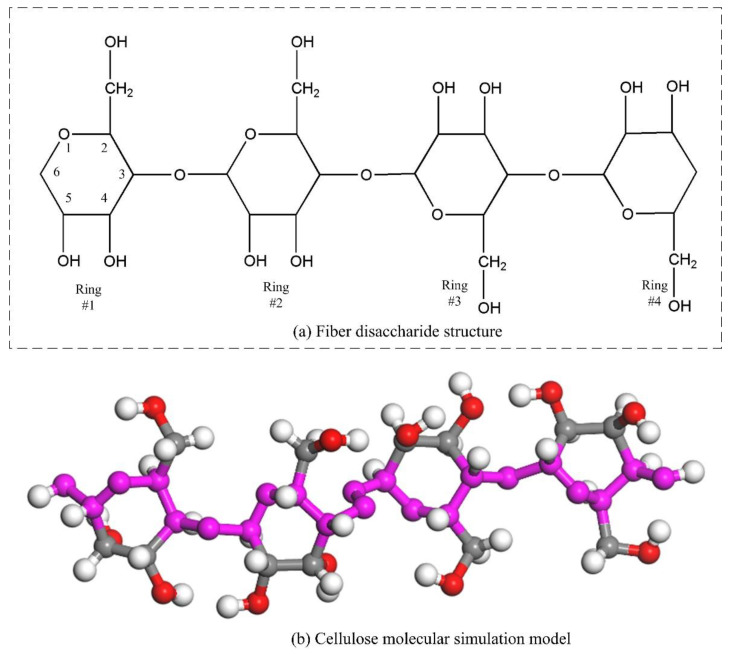
Molecular model diagram based on molecular structure. (**a**) Fiber disaccharide structure, (**b**) Cellulose molecular simulation model.

**Figure 6:**
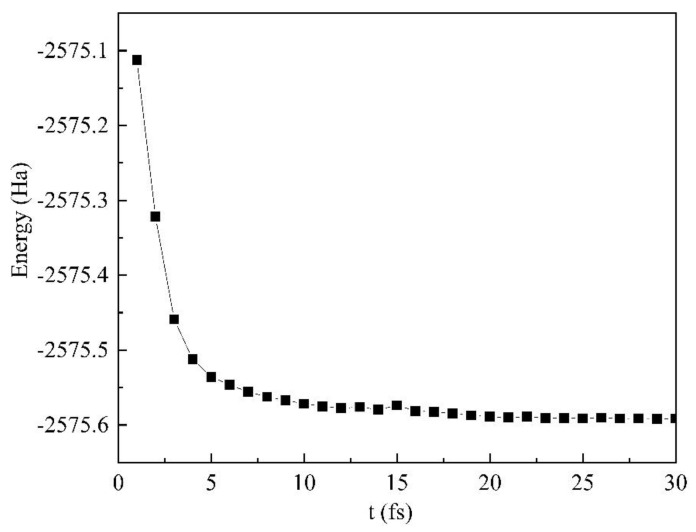
Energy changes in the structural optimization process.

In the simulation process, the degree of polymerization of cellulose molecule is set to 2, and the molecular structure of cellobiose is shown in Figure 5a. The established cellulose molecule is geometrically optimized, as shown in Figure 5b. The purpose of geometric optimization is to make the molecule reach a stable configuration and a steady-state. As shown in Figure 6, the molecular energy has reached a ready state, allowing accurate calculation in molecular dynamics simulation.

In the work, the molecular dynamics module, the DMOL module, was used to extract samples from the system composed of different states of the molecular system, in order to calculate the configuration integral of the system. Based on the results of the configuration integral, the thermodynamic quantities and other macroscopic properties of the system were further calculated. The theory used in this module is Density Functional Theory (DFT), which indicates that all state properties are functionals of the charge density [21,22,23].

The distribution of the electron cloud is the wave function. The electron cloud of the atom is the wave function of an atom. The electron cloud of a molecule is the linear combination of all atomic electron clouds, and the wave function of an atom is the combination of all basic functions. In this module, the selection of the basic group is very important. The DNP basis set was selected, which adopts Double Numerical plus polarization (DNP), which has high accuracy.

According to molecular orbital theory and Huckel molecular orbital method (HMO), the molecular properties are determined by the orbitals near LUMO and HOMO. The LUMO and HOMO energy levels reflect the electrophilicity and nucleophilicity of the molecule, respectively. The energy gap of LUMO and HOMO reflects the ability of electron transition from occupied orbit to empty orbit. The smaller the energy gap, the more easily the electron leap occurs. Large numbers of electrons jumping from low to high energy level causes greater chemical activity of molecules.

**Figure 7:**
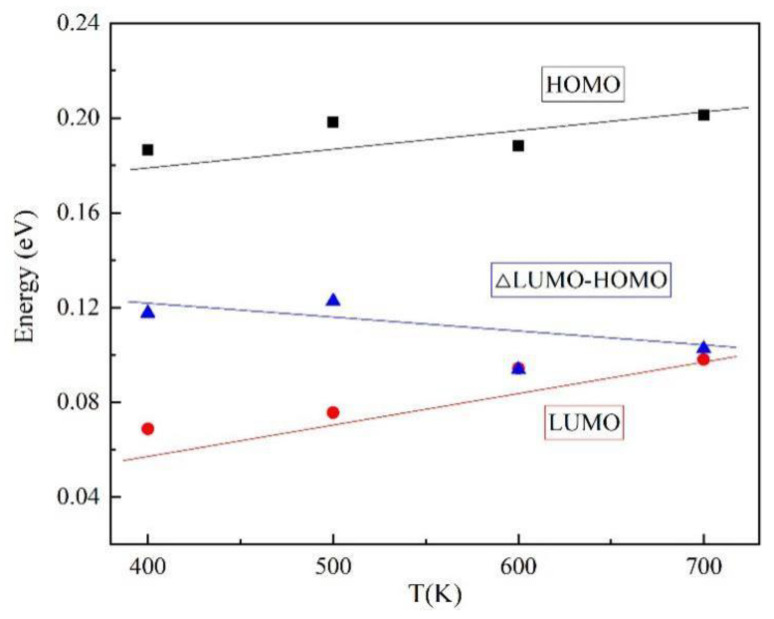
Orbital energy at different simulated temperatures.

As shown in Table 3 and Figure 7, the total molecular energy changes little at different simulated temperatures, the highest orbit occupies a relatively stable position, the lowest orbitals have a certain tendency to increase, the energy gap decreases, and the activity of the molecule increases. This indicates that the molecular thermal movement of insulation paper is more intense and the stability of insulation paper decreases as the transformer operating temperature increases.

**Figure 8:**
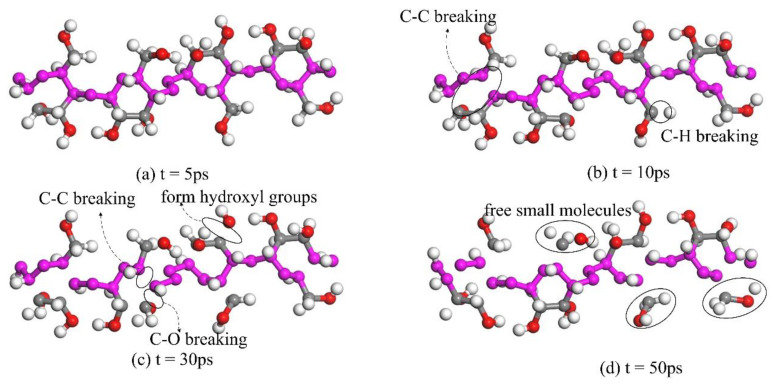
Thermal cracking process of cellulose molecules.

Figure 8 shows the dynamic change process of cellulose molecules at 400 K. With the change in the number of simulation frames, the whole molecule twisted and vibrated, which led to the tensile fracture of chemical bonds and the formation of free small molecules, as shown in Table 4 [24]. The stability of molecules was damaged and the degree of polymerization decreased [25,26].

**Figure 9:**
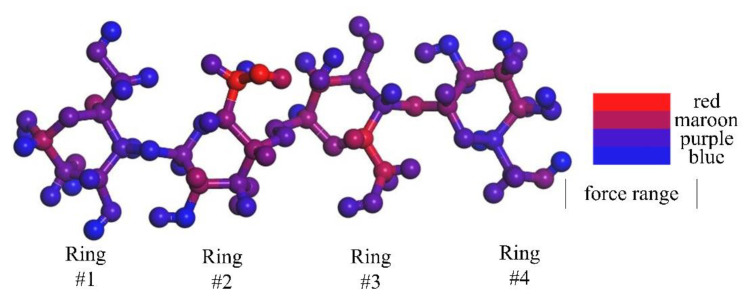
Force distribution of cellulose molecules.

To study the molecular microscopic force patterns and establish the defect model, the molecular dynamics at 400 K are analyzed and the forces of the atoms are marked with color. The force on each molecular position is shown in Table 5. As can be seen in Figure 9, red is the place with the most stress, blue is the place with low stress, and the middle is the transition.

**Figure 10:**
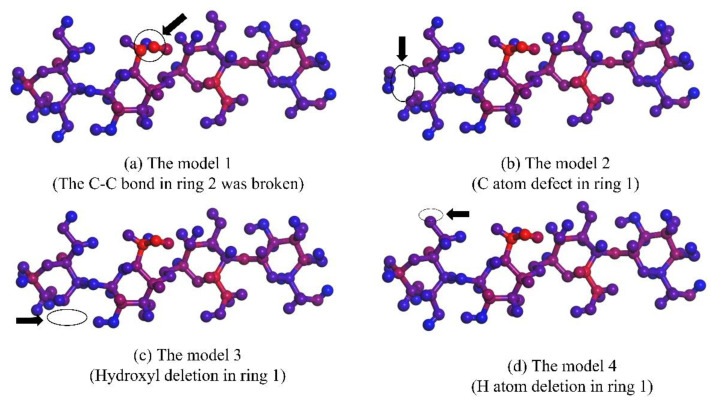
Cellulose molecular defect model.

Red is shown in Figure 9 C–O on ring #2, C–C on ring #3. Maroon: C atom at position 6 on ring #1, H atom attached to C atom at position 2, C atoms at positions 2 and 3 and their C–C bonds on ring #2, C–H at position 5, C atom at position 1, and C–O at position 5–6 on ring #3, C–C bond at positions 2–3 and C atom at position 6 on ring #4. The other position with small force marked with blue or purple is not used for research. On this basis, the defect models were constructed with the loss of atoms or bonds mentioned above, as shown in Figure 10.

Electron affinity can be regarded as the energy change of adding electrons or extracting holes on the molecule, which indicates the difficulty of an electron escaping from the material. The smaller the electron affinity, the easier the electrons escape from the material. The calculation of electron affinity is shown in the equation.

(6) $$E_{A}=\left|E\left(R_{E}\right)-E^{\prime }(R_{E})\right|$$

where, *E*(*R_E_*) is the total energy of a neutral molecule in a stable configuration, while *E*′(*R_E_*) is the total energy of a negatively charged anion molecule in a stable configuration. The trap depth of cellulose can be characterized by subtracting the electron affinity of the complete molecular model from the electron affinity of the defective molecular model.

It can be seen in these models that the closer the color is to red in Table 6, the larger the trap energy level is. The highest trap energy level, 0.96 eV, is found in the model with the breaking of C–C bonds in ring #2; the lowest trap energy level, 0.4 eV, is found in the model with the absence of H atoms in ring #1.

The trap energy level at the non-aging stage is about 0.54 eV, which indicates that the cellulose is intact at this stage and a small amount of H atom deletion exists. The trap energy level at the middle stage of aging is about 0.73 eV and some C atoms are missing. The trap energy level at the end of aging is about 0.92 eV, which is caused by the breaking of chemical bonds. The greater the force on the molecule, the more easily the structure of the molecule will be destroyed. The protection of these places should be strengthened to maintain the stability of the molecule.

## 5. Conclusions

In conclusion, the trap parameters of oil-paper insulation under thermal aging were studied based on the experiment and molecular simulation. The trap characteristic parameters are extracted through space charge curves, and the trap change mechanisms are explained by molecular simulation.

(1) The experimental results show that the trap energy levels range from 0.4 eV to 0.92 eV, with 0.54 eV at the beginning of aging (DP1490), 0.73 eV at the middle of aging (DP787), and 0.92 eV at the end of aging (DP311).

(2) The simulation results show that with the increase in aging time, the whole cellulose molecule was twisted and vibrated, and gradually cracked into small molecular fragments. A large number of small molecules were produced when the simulation time reached 100 ps. The trap energy level characterized by affinity potential was also between 0.4 eV and 0.96 eV, which corresponded to the trap energy level extracted from the experiment.

(3) In the defect model established by the force, the greater the force on the molecular structure, the more intense the molecular motion, and the larger the trapped energy level. The trap energy level of the model of C–C fracture of ring 2 is the largest, which is 0.96 eV, and the trap energy level of the defect model established by the absence of H atom is the smallest, which is 0.4 eV. The trap energy level is 0.54 eV in the early stage of aging, which indicates that the cellulose is intact at this stage and a small amount of H atom deletion occurs. The trap energy level is 0.73 eV at the middle of aging, which is due to the loss of some C atoms in cellulose molecules. The trap energy level is 0.92 eV at the end of aging, which is caused by the breaking of chemical bonds. The greater the force on the molecule, the more easily the structure of the molecule will be destroyed. The protection of these places should be strengthened to maintain the stability of the molecule.

## Figures and Tables

**Table 1:** Degree of polymerization of samples with different aging times.

**Aging Time (h)**	0	96	360	720	1200
**Degree of Polymerization (DP)**	1490	787	584	510	311

**Table 2:** Morphologies of samples with different aging times.

**Degree of Polymerization (DP)**	1490	787	311
**Insulationoil**	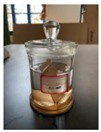	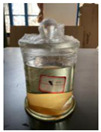	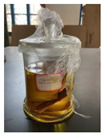
**SEM of Insulation paper**	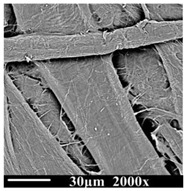	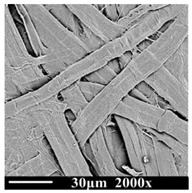	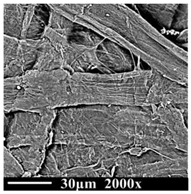

**Table 3:** Molecular orbital occupation energy scale.

Temperature	Total Energy (Ha)	HOMO (eV)	LUMO (eV)	ΔLUMO−HOMO (eV)
400 K	−2594.71	0.186	0.068	0.118
500 K	−2594.66	0.198	0.076	0.123
600 K	−2594.62	0.188	0.094	0.094
700 K	−2594.62	0.201	0.098	0.102

**Table 4:** Number of small molecules after 100 ps.

**Molecular Type**	H^+^	OH^−^	CH_2_O	C_2_H_4_O_2_
**Number**	8	3	6	1

**Table 5:** The force exerted on different atoms.

Atomic Position	X-Axis Force (kcal/mol/Å)	Y-Axis Stress (kcal/mol/Å)	Z-Axis Force (kcal/mol/Å)
C atom of ring 2 (red)	83.134	39.380	28.193
O atom on ring 2 (red)	74.811	36.516	28.636
No. 1 C atom in No. 3 ring	14.527	33.439	68.200
H atom on ring 1 (blue)	5.178	8.154	1.743

**Table 6:** Trap level of different cellulose defect models.

Model	Neutral Molecule (Ha)	Anion (Ha)	Intact Molecule (eV)	Trap Level (eV)
Model 1	2594.196	2594.348	0.118	0.96
Model 2	2594.346	2594.498	0.118	0.92
Model 3	2594.536	2594.674	0.118	0.56
Model 4	2594.434	2594.566	0.118	0.40

## Data Availability

The data presented in this study are available on request from the corresponding author.
